# Using Modified Sample Entropy to Characterize Aging-Associated Microvascular Dysfunction

**DOI:** 10.3389/fphys.2016.00126

**Published:** 2016-04-12

**Authors:** Fuyuan Liao, Yih-Kuen Jan

**Affiliations:** ^1^Department of Biomedical Engineering, Xi'an Technological UniversityXi'an, China; ^2^Rehabilitation Engineering Lab, Department of Kinesiology and Community Health, University of Illinois at Urbana-ChampaignChampaign, IL, USA; ^3^Computational Science and Engineering Program, University of Illinois at Urbana-ChampaignUrbana, IL, USA

**Keywords:** aging, skin blood flow, complexity, sample entropy, blood flow oscillations

## Abstract

Cutaneous microvascular function can be assessed by skin blood flow (SBF) response to thermal stimuli. Usually, the activities of the regulatory mechanisms are quantified by means of spectral analysis of the response. However, spectral measures are unable to characterize the nonlinear dynamics of SBF signal. Sample entropy (SampEn) is a commonly used nonlinear measure of the degree of regularity of time series. However, SampEn value depends on the relationship between the frequency of the studied dynamics and sampling rate. Hence, when time series data are oversampled, SampEn may give misleading results. We modified the definition of SampEn by including a lag between successive data points of the vectors to be compared to address the oversampled issue. The lag could be chosen as the first minimum of the auto mutual information function of the time series. We tested the performance of modified SampEn using simulated signals and SBF data in the young and old groups. The results indicated that modified SampEn yields consistent results for different sampling rates in simulated data, but SampEn cannot. Blood flow data showed a higher degree of regularity during the maximal vasodilation period as compared to the baseline in both groups and a higher degree of regularity in the older group as compared to the young group. Furthermore, our results showed that during the second peak the more regular behavior of blood flow oscillations (BFO) is mainly attributed to enhanced cardiac oscillations. This study suggests that the modified SampEn approach may be useful for assessing microvascular function.

## Introduction

The aging process causes both structural and functional changes in the cardiovascular system (Marin, 1995). These changes may attenuate microvascular reactivity in response to environmental stimuli (Holowatz et al., 2010). It has been well known that elevated skin temperature is a major cause factor of pressure ulcers in older people due to prolonged sitting or bedrest (Fisher et al., 1978; Jan and Brienza, 2006). The increased skin temperature raises the metabolic demands of local cells and tissues on the order of 10% for every 1°C (Jan et al., 2012). The inability of skin microcirculation to remove excessive heat and meet the metabolic needs by increasing skin blood flow (SBF) can result in tissue ischemia of weight-bearing soft tissues, thus increasing risk of pressure ulcers (Jan et al., 2009; Liao et al., 2013). Therefore, SBF response to local heating may be used to assess the risk of pressure ulcers in older people.

SBF response to local heat has been found to depend on the temperature, rate, and duration of heat (Minson et al., 2001; Jan et al., 2009; Johnson and Kellogg, 2010). When local temperature is rapidly increased to 42°C and maintained at that level, SBF shows a biphasic response characterized by an initial increase (the first peak) followed by a brief nadir, and then a slowly developing rise to a plateau (the second peak; Minson et al., 2001). The first peak is mediated predominantly by local sensory nerves, and the second peak/plateau is mediated predominantly by nitric oxide (Johnson and Kellogg, 2010; Minson, 2010). In this context, cutaneous vascular conductance and biphasic thermal index are commonly used to characterize the response (Minson et al., 2002; Jan et al., 2009). Furthermore, more sophisticated techniques such as wavelet-based spectral analysis have been utilized to explore the underlying mechanisms of the response (Jan et al., 2009). Wavelet analysis of blood flow oscillations (BFO) in human skin has revealed six frequencies in the frequency area below 2.0 Hz (Stefanovska et al., 1999; Geyer et al., 2004; Kvandal et al., 2006). The oscillations around 1.0 and 0.3 Hz are originated from heart beats and respiration, respectively; the oscillations around 0.1, 0.04, 0.01, and 0.007 Hz are associated with the myogenic activity of vascular smooth muscle, the neurogenic activity of the vessel wall, and two different mechanisms of vascular endothelial function, respectively (Stefanovska et al., 1999; Geyer et al., 2004; Kvandal et al., 2006). It is hypothesized that the oscillations around 0.01 and 0.007 Hz involve nitric oxide and endothelium-derived hyperpolarizing factors, respectively {Stefanovska, 2007 #99}. Recent studies by our group have shown that decreased vasodilation with age during local heating is associated with diminished metabolic, neurogenic, and myogenic activities (Jan et al., 2009).

Although wavelet analysis of BFO provides a straightforward interpretation of the active state of the regulatory mechanisms of SBF, spectral measures are unable to characterize the nonlinear dynamics of BFO {Liao et al., 2013 #14}. Nonlinear properties of BFO have been probed by applying methods based on fractal theory and nonlinear dynamics (Humeau et al., 2008; Liao et al., 2010; Shiogai et al., 2010; Jan et al., 2011; Liao and Jan, 2011). Among these methods, sample entropy (*S*_*E*_) developed by Richman and Moorman (2000) can be easily applied to short and noisy time series data (Richman and Moorman, 2000). Although *S*_*E*_ has been demonstrated to have important advantages over approximate entropy (*A*_*E*_) (Ping and Johnson, 1992), we observed that for SBF data, *S*_*E*_ also depends on the relationship between the frequency of the studied dynamics and the sampling rate. As a consequence, when data series is oversampled, i.e., the sampling frequency is much higher than the frequency of the studied time series, *S*_*E*_ may give misleading results.

A possible approach for resolving the above problem is to include a lag between successive data points of the vectors to be compared. This idea has been proposed by Richman et al. (2004) and Govindan et al. (2007) but, to our knowledge, has not been applied to SBF data. Govindan et al. (2007) suggested that since it is difficult to choose an optimal lag for long-range correlated data, one may consider the increment of the data, for which the time lag will be 1. However, the increment basically contains the high frequency components of the data. Such a transform may not be appropriate for SBF data, because the low frequency components of BFO are associated with the local control mechanisms of SBF (Stefanovska et al., 1999; Kvandal et al., 2006). We found that for both simulated deterministic and SBF data, the first minimum of the automutual information (MI) function is a good choice of the lag.

In this paper, we modify the *S*_*E*_ algorithm by using time-lagged vectors in the calculation of the conditional probability that two vectors are similar for *m* points remaining similar at the next point. The lag was chosen as the first minimum of the MI function. We systematically test the performance of the modified *S*_*E*_ approach using simulated signals, including sine wave and time series from Rössler attractor, and SBF data. Next, we applied *S*_*E*_ and modified *S*_*E*_ to SBF data from healthy young and older adults during the baseline and local heating-induced second peak period. We hypothesized that modified *S*_*E*_ would be able to reflect the degree of regularity of BFO regardless of sampling rate.

## Methods

### Subjects

Seventeen healthy young subjects and 13 older subjects were recruited into this study. The young group included 8 males and 9 females, age 25 ± 5.6 years (mean ± SD), and body mass index 23.6 ± 2.8 kg/m^2^; the older group included 6 males and 7 females, age 72.3 ± 5.8 years, and body mass index 25.1 ± 2.4 kg/m^2^. The exclusion criteria included any diagnosed cardiopulmonary diseases, smoking history, or use of any medication that may affect cardiopulmonary function. All participants gave written informed consent to participate in this study, which was approved by the Institutional Review Board of the University of Oklahoma for human subject research.

### Data collection

**Figure 1:**
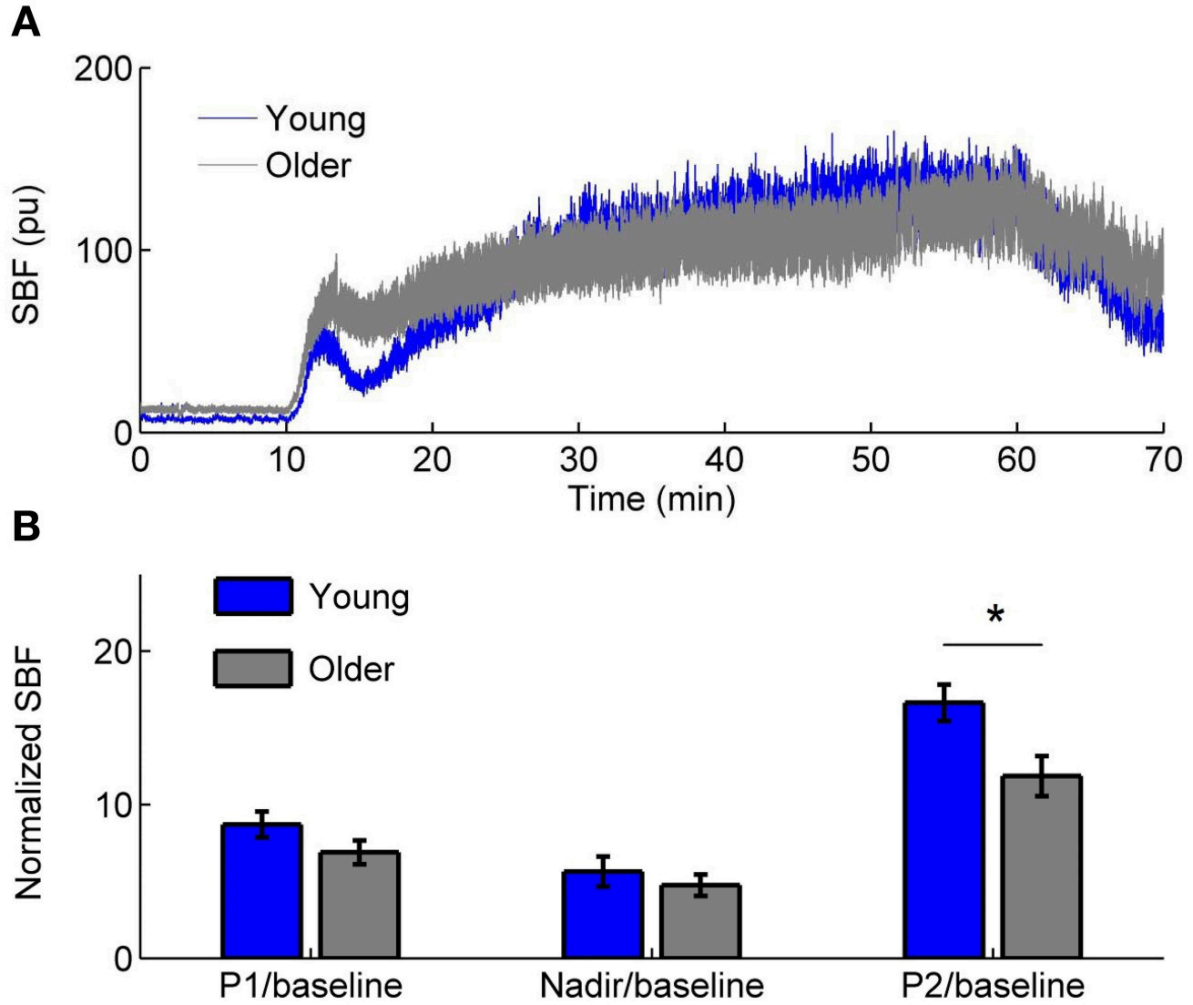
**(A)** Sacral skin blood flow (SBF) in response to a rapid local heating to 42°C in a young subject and an older subject. pu, perfusion unit. **(B)** Normalized SBF at the initial peak (P1), nadir, and second peak (P2, 51–60 min). Values are means± standard errors. ^*^ indicates a significant difference between two groups (*p* < 0.05, Wilcoxon rank sum test).

The experiments were conducted in a university research laboratory. Room temperature was maintained at 24 ± 2°C. All subjects stayed in the lab at least 30 min prior to the experiment to become acclimated to the room temperature and to achieve a steady baseline blood flow. When the subject was in a prone position, a combined probe of heating and laser Doppler flowmetry (LDF) (Probe 415-242 & PF5010, Perimed AB) was used to heat the sacral skin to 42°C in 2 min and to maintain that temperature level (Jan et al., 2009). Skin blood flow was recorded by LDF at a sampling rate of 32 Hz. The protocol included a 10-min baseline, a 50-min heating period, and a 10-min recovery period. Figure 1 shows typical SBF responses in a young subject and an older subject and the results of normalized SBF at the initial peak, nadir, and second peak in two groups.

### Sample entropy algorithm

Given a time series of length *N*, {*x*(*i*), *i* = 1, …, *N*}, the sample entropy algorithm is computed as follows (Richman and Moorman, 2000).

1. Consider vectors of length m: ***x***_*m*_(*i*) = {*x*(*i* + *k*), 0 ≤ *k* ≤ *m* − 1}, 1 ≤ *i* ≤ *N* − *m*. The distance between two such vectors is defined as *d*[***x***_*m*_(*i*), ***x***_*m*_(*j*)] = *max*?{|*x*(*i* + *k*) − *x*(*j* + *k*)|, 0 ≤ *k* ≤ *m* − 1}.
2. For a given vector ***x***_*m*_(*i*), let $n_{i}^{m}\left(r\right)$ be the number of vectors ***x***_*m*_(*j*) that are within a tolerance *r* of ***x***_*m*_(*i*), i.e., *d*[***x***_*m*_(*i*), ***x***_*m*_(*j*)] ≤ *r*, where *j* ≠ *i*. Thus, $C^{m}\left(r\right)=\frac{1}{N-m}\overset{N-m}{\underset{i=1}{\sum }}n_{i}^{m}\left(r\right)$ represents the probability that any vector ***x***_*m*_(*j*) is within r of the vector ***x***_*m*_(*i*).
3. Likewise, calculate the probability that any two vectors *x*_*m*+1_(*i*) and *x*_*m*+1_(*j*) are within r of each other, *C*^*m*+1^(*r*), where *j* ≠ *i*.
4. Finally, sample entropy is defined as
  (1) $$\begin{array}{l} S_{E}\left(m,r\right)=\underset{N\to \infty }{\lim}-ln\frac{C^{m+1}\left(r\right)}{C^{m}\left(r\right)}, \end{array}$$
  which is estimated by the statistic
  (2) $$\begin{array}{l} S_{E}\left(m,r,N\right)=-ln\frac{C^{m+1}\left(r\right)}{C^{m}\left(r\right)}. \end{array}$$

The tolerance *r* is usually set to be *r* × SD, where SD is the standard deviation of the time series. Throughout this work, all time-series data were normalized to have SD=1 when computing entropy.

In the *S*_*E*_ algorithm, the constraint condition *j* ≠ *i* excludes self-matches, which are included in the *A*_*E*_ algorithm. Thus, *S*_*E*_ has reduced bias compared to *A*_*E*_ (Richman and Moorman, 2000). Furthermore, *S*_*E*_ is largely independent of record length and shows relative consistency under conditions where *A*_*E*_ does not (Richman and Moorman, 2000). However, we observed that for temporally correlated data, *S*_*E*_ is dependent on the relationship between the frequency of the studied time series and the sampling rate. To demonstrate this point, we calculated *S*_*E*_ for numerically simulated deterministic signals and SBF data. As for deterministic signals, we considered the sine wave *sin*(2π · 0.1*t*) and the variable *x*_1_ of Rössler attractor

(3) $$\begin{array}{l} dx_{1}∕dt=-x_{2}-x_{3},\\ dx_{2}∕dt=x_{1}+0.2x_{2},\\ dx_{3}∕dt=0.2+x_{3}\left(x_{1}-5.7\right). \end{array}$$

**Figure 2:**
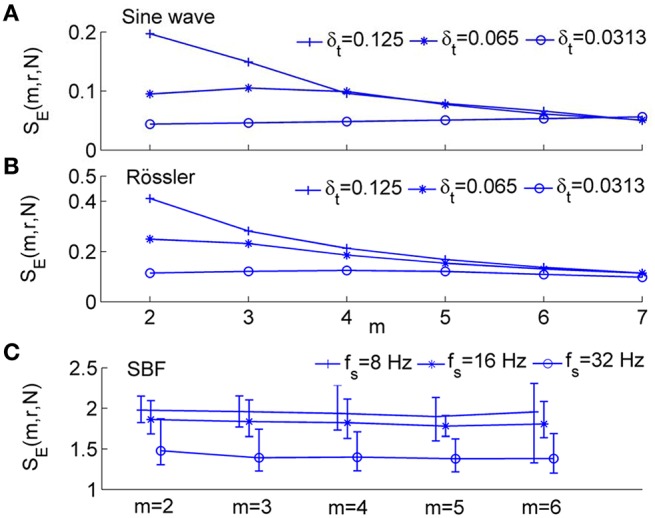
**Sample entropy, ***S***_***E***_(***m, r, N***), for numerically simulated signals and SBF data, where ***r*** = 0.2 and ***N*** = 4800. (A)** Values of *S*_*E*_(*m, r, N*) for *sin*(2π·0.1*t*) sampled at δ_*t*_ = 0.125, 0.0625, and 0.0313 s, respectively, where δ_*t*_ is the sampling interval. **(B)** Values of *S*_*E*_(*m, r, N*) for the variable *x*_1_ of Rössler attractor (Equation 3) sampled at δ_*t*_ = 0.125, 0.0625, and 0.0313 s, respectively. **(C)** Values of *S*_*E*_(*m, r, N*) for SBF signals from all the subjects during 1–10 min (baseline). The sampling rate *f*_*s*_ was chosen as 8, 16, and 32 Hz, respectively. The results are shown as mean values, 5th percentiles, and 95th percentiles.

As shown in Figure 2, for the sine wave and Rössler attractor, *S*_*E*_ values can be different for δ_*t*_ = 0.0313, δ_*t*_ = 0.0625, and 0.125 s, where δ_*t*_ is the sampling interval (Figures 2A,B). With increasing *m* values, the difference in *S*_*E*_ due to different sampling intervals becomes smaller. For the SBF signals shown in Figure 1A during 1–10 min (older subject), *S*_*E*_ values are distinctly different for different sampling rates (Figure 2C).

The dependence of *S*_*E*_ on sampling rate is mainly attributed to the correlation in time series. Given a correlated signal, when data points are sampled at a higher sampling rate, values of successive data points are more close to each other and hence two vectors within r for m points likely remain within r at the next point. In this case, the conditional probability *C*^*m*+1^(*r*)∕*C*^*m*^(*r*) (Equation 2) will be assigned a larger value and thus a smaller value of *S*_*E*_ will be obtained. In contrast, a lower sampling rate leads to a larger value of *S*_*E*_. Because physiological signals, e.g., SBF signals, usually possess long-range correlations, the dependence of *S*_*E*_ on sampling rate may cause a series of problems. One problem is that different sampling rates can lead to different interpretations of the same process in terms of “regularity.” Later we will show that if SBF data are oversampled, *S*_*E*_ may not be able to reflect changes in spectrum of BFO in the frequency area below 2 Hz.

### Modified sample entropy algorithm

The influence of sampling rate on *S*_*E*_ estimation may be eliminated by using a lag between the successive data points of the vectors to be compared. This procedure is similar to the reconstruction of dynamics from a time series for computing correlation dimension and Lyapunov exponents (Stam, 2005). We make two alterations to the original *S*_*E*_ algorithm. First, we use time-lagged vectors, which have the following form

(4) $$\begin{array}{l} x_{m}^{\tau }\left(i\right)=\left\{x\left(i+k\tau \right),\;0\leq k\leq m-1\right\},1\leq i\leq N-m\tau , \end{array}$$

where τ is the lag. The condition1 ≤ *i* ≤ *N* − *mτ* ensures that $x_{m+1}^{\tau }\left(i\right)$ will be defined when *i* = *N* − *mτ*. Second, when counting the number of $x_{m}^{\tau }\left(j\right)$ that are within *r* of $x_{m}^{\tau }\left(i\right)$, we consider only the vectors $x_{m}^{\tau }\left(j\right)$ satisfying |*j* − *i*| > τ; likewise, for each vector $x_{m+1}^{\tau }\left(i\right)$, we consider only the vectors $x_{m+1}^{\tau }\left(i\right)$ satisfying |*j* − *i*| > τ. This constraint condition is aimed to minimize the influence of the correlation on entropy estimation. We denote the modified sample entropy as *S*_*E*_(*m, r*, τ, *N*).

A critical step in the *S*_*E*_(*m, r*, τ, *N*) algorithm is to determine a lag τ. In previous studies on this topic, time lag has been chosen as the first zero crossing of the autocorrelation function *C*(τ) (Cellucci et al., 2003), the time point where the autocorrelation function drops to 1∕*e* or 1 − 1∕*e* of its initial value (Rosenstein et al., 1993; Stam, 2005), or the first minimum of the MI function MI(τ) (Kantz and Schreiber, 1997). The first zero crossing of *C*(τ), τ_0_, means that, on average, the observation *x*(*i*) and *x*(*i* + τ_0_) will be lineally independent. Abarbanel (1996) argued that the first minimum of MI(τ) is a more appropriate choice of the lag because MI(τ) can be viewed as a nonlinear analog of *C*(τ). Bradley and Kantz {Bradley and Kantz, 2015 #100} suggested that the first minimum of MI(τ) represents a more general form of independence compared with the first zero crossing of *C*(τ). On the other hand, *C*(τ) often results in overestimates of the time delay when applied to nonstationary time series {Clemson and Stefanovska, 2014 #45}. Cellucci et al. {Cellucci et al., 2003 #101} performed a comparative study of embedding methods and concluded that the best value of delay can be identified by *MI*(τ).

**Figure 3:**
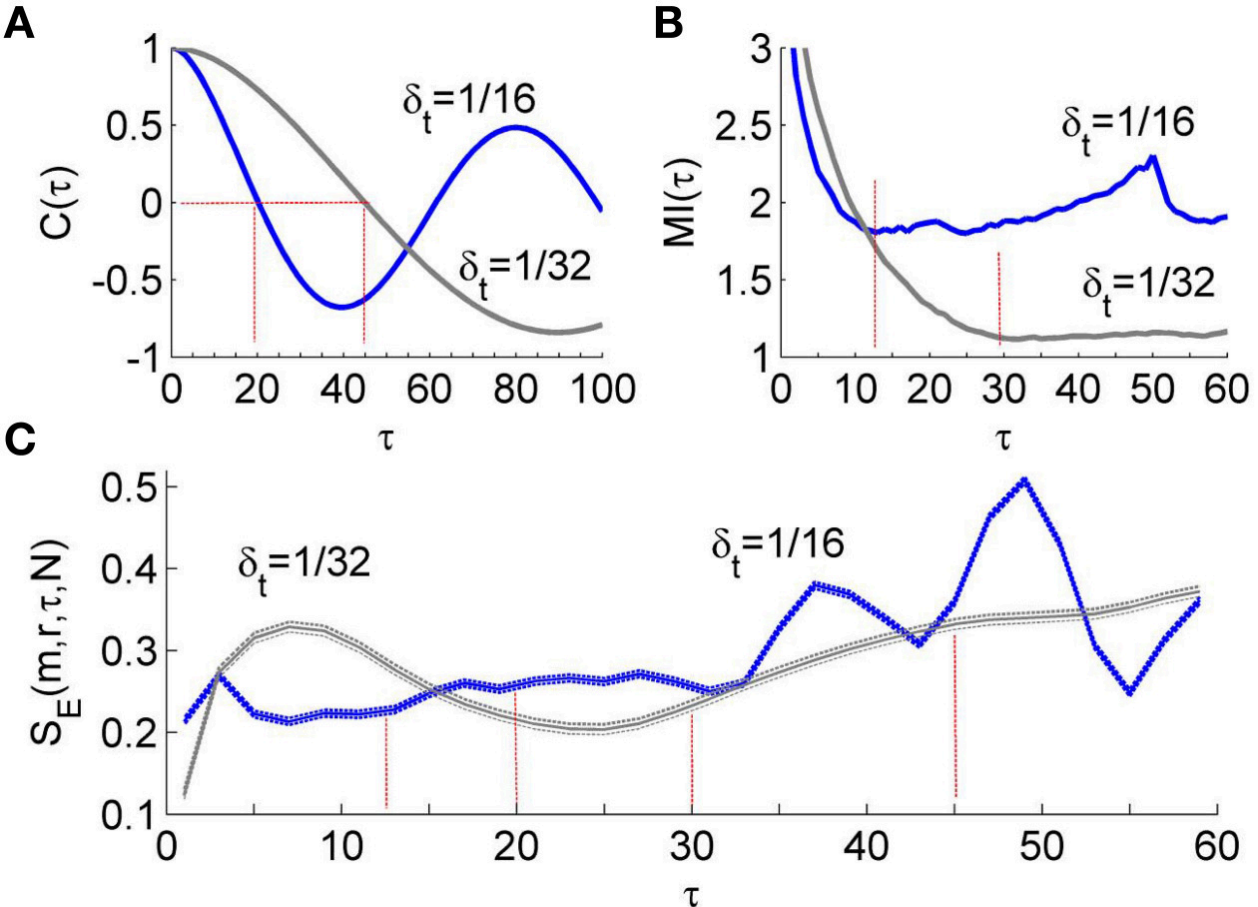
**(A)** Autocorrelation function *C*(τ) of the time series derived from simulated Rössler attractor (Equation 3). The Rössler attractor was simulated 10 times for each of the step sizes δ_*t*_ = 1∕16 and δ_*t*_ = 1/32 s using different initial conditions. The time series were composed of 4800 samples from the steady-state portion of the variable *x*_1_. The first zero crossing of *C*(τ) is around 20 for δ_*t*_ = 1/16 and 45 for δ_*t*_ = 1/32. **(B)** Mutual information function MI(τ) of the time series. The first minimum of MI(τ) is around 12 for δ_*t*_ = 1/16 and 30 for δ_*t*_ = 1/32. **(C)** Values of *S*_*E*_ presented as mean ± standard deviations. Using the first minimum of MI(τ), *S*_*E*_ yields almost identical values for different step sizes, whereas when using the first zero crossing of *C*(τ) as the lag τ, *S*_*E*_ yields distinctly different values for different step sizes.

To compare the performance of the first minimum of MI(τ) and that of the first zero crossing of *C*(τ) in the calculation of *S*_*E*_, we performed the following experiment. We simulated the Rössler attractor (Equation 3) 10 times for each of the step size 1/16 and 1.32 s using different initial conditions. Then we analyzed the time series composed of 4800 samples from the steady-state portion of the variable *x*_1_. The results are shown in Figure 3. Using the first minimum of MI(τ) as the lag τ, *S*_*E*_ yields almost identical values for different step sizes, whereas when using the first zero crossing of *C*(τ) as the lag τ, *S*_*E*_ yields distinctly different values for different step sizes. These results suggest that the first minimum of MI(τ) is a more appropriate choice of the delay for computing sample entropy.

**Figure 4:**
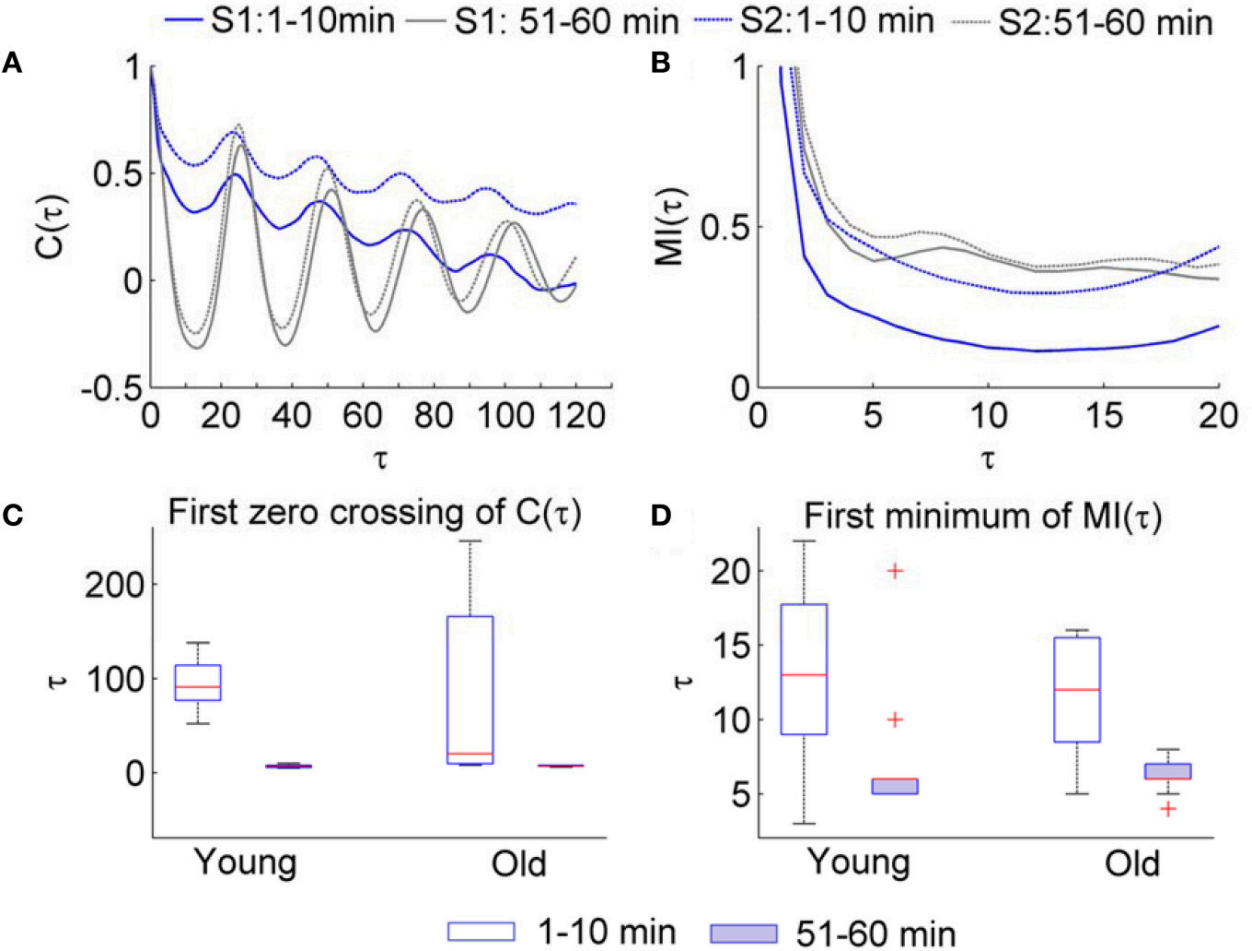
**Values of τ for the SBF signals determined by the autocorrelation function ***C***(τ) and mutual information function MI(τ)**. The sampling rate was 32 Hz. **(A)** Examples of *C*(τ) of SBF signals during 1–10 min and 51–60 min. S1and S2 denote two subjects. **(B)** Examples of MI(τ) of the SBF signals during 1–10 min and 51–60 min. **(C)** Values of τ determined as the first zero crossing of *C*(τ). **(D)** Values of τ determined as the first minimum of MI(τ).

Regarding SBF signals, we observed that during the baseline period (1–10 min), the first zero crossing of *C*(τ) yields a very large value (Figure 4A) or does not exist at all; during the second peak (51–60 min), it yield a much smaller value (Figure 4C). This observation suggests that the lag τ estimated by the first zero crossing of *C*(τ) is dubious when applied to SBF signals. Unlike *C*(τ), the first minimum of MI(τ) yields a moderate value during the baseline and a smaller value during the second peak (Figures 4B,D). On the other hand, because the optimal lag in time is a constant, the first minimum of MI(τ) of the data series is usually proportional to sampling rate. This feature ensures a constant degree of dependence between successive data points of the vectors to be compared regardless of sampling rate. In addition, if the first minimum of the MI function is unobvious when the signal is sampled at a higher sampling rate, e.g., 32 Hz, it would be easier to determine the lag by checking the MI(τ) of a downsampled version of the signal.

### Validity of modified sample entropy

**Figure 5:**
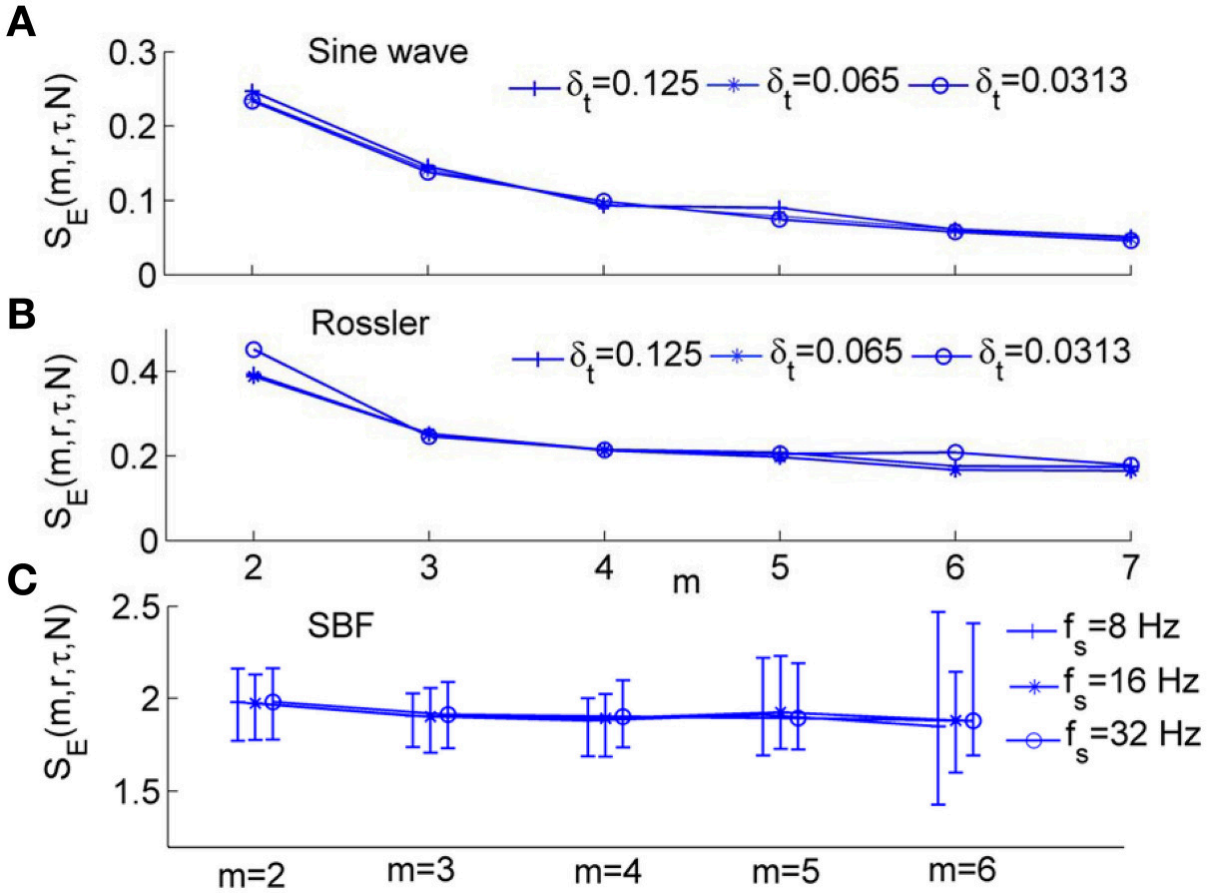
**Modified sample entropy, ***S***_***E***_(***m, r***, τ, ***N***), for numerically simulated signals and SBF data, where ***r*** = 0.2 and ***N*** = 4800. (A)** Values of *S*_*E*_(*m, r*, τ, *N*) for sin(2π·0.1*t*) sampled at δ_*t*_ = 0.125 and 0.0625, and 0.0313 s, respectively. The values of τ are 2, 4, and 8 respectively. **(B)** Values of *S*_*E*_(*m, r*, τ, *N*) for the variable *x*_1_ of Rössler attractor (Equation 3) sampled at δ_*t*_ = 0.125, 0.0625, and 0.0313 s, respectively. The values of τ are 8, 16, and 32 respectively. **(C)** Values of *S*_*E*_(*m, r*, τ, *N*) for SBF signals from all the subjects during 1–10 min. The sampling rate *f*_*s*_ was chosen as 8, 16, and 32 Hz, respectively. The parameter τ = 12 was used based on the distribution of τ values for all the subjects (Figure 4D). The results are shown as means, 5th percentiles, and 95th percentiles.

We systematically evaluated the performance of *S*_*E*_(*m, r*, τ, *N*) using both numerically simulated and SBF data. We first examined whether *S*_*E*_(*m, r*, τ, *N*) depends on sampling rate. As shown in Figures 5A,B, for the sine wave and Rössler attractor, *S*_*E*_(*m, r*, τ, *N*) is independent of sampling rate for m from 2 to 8. For SBF signals during 1–10 min, *S*_*E*_(*m, r*, τ, *N*) is independent of sampling rate for m ranging from 2 to 5 (Figure 5C). We noted that when *m* > 5, the probabilities *C*^*m*^(*r*) and *C*^*m*+1^(*r*) (Equation 2) are too small that *S*_*E*_(*m, r*, τ, *N*) becomes unreliable.

**Figure 6:**
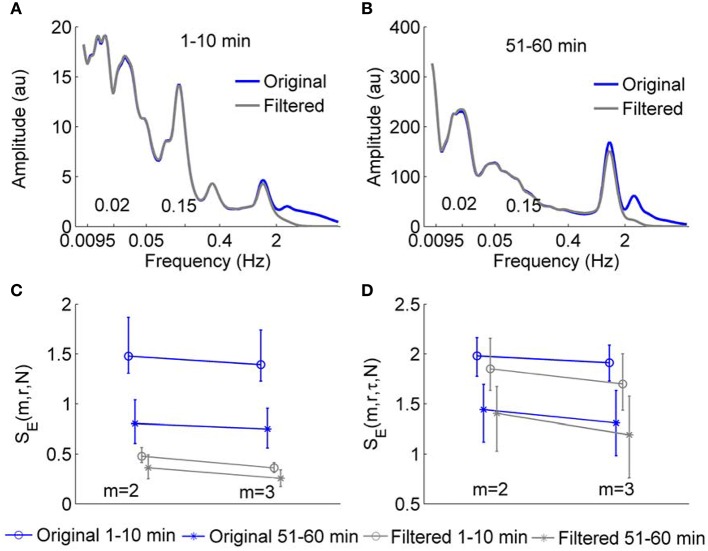
**(A,B)** Wavelet amplitude spectra of a SBF signal during 1–10 min and 51–60 min before and after filtering. Here, wavelet amplitudes are averaged absolute values of the wavelet transform over time. The Morlet wavelet was used to implement continuous wavelet transforms. **(C,D)** Values of *S*_*E*_(*m, r, N*) and *S*_*E*_(*m, r*, τ, *N*) for 1–10 min and 51–60 min segments of SBF signals from all the subjects before and after removing the components with frequencies higher than 2 Hz. The sampling rate was 32 Hz and the parameter *r* = 0.2, τ = 12, and *N* = 4800 were used. The results are shown as means, 5 percentiles, and 95 percentiles.

We next tested whether *S*_*E*_(*m, r*, τ, *N*) and *S*_*E*_(*m, r, N*) are able to reflect changes in structural properties of BFO with frequencies below 2 Hz. As mentioned earlier, SBF signals in human skin have been found to contain at least six characteristic frequency components in the frequency area below 2 Hz, each of which corresponds to a specific underlying mechanism (Stefanovska et al., 1999; Kvandal et al., 2006). We performed the following experiment. For the SBF signals sampled at 32 Hz, we calculated *S*_*E*_(*m, r*, τ, *N*) and *S*_*E*_(*m, r, N*) for the 1–10 min and 51–60 min segments before and after removing the components with frequencies higher than 2 Hz. The parameters *m* = 2 and 3 and *r* = 0.2 were used. Figure 6 presents the results for the signals from all the subjects. For the original signal, *S*_*E*_(*m, r, N*) of the 51–60 min segments is much lower than that of the 1–10 min segments, whereas for the filtered signals, values of *S*_*E*_(*m, r, N*) of the 1–10 min segments are similar to those for the 51–60 min segments (Figure 6C). However, by using wavelet analysis, it can be seen that the 1–10 min and 51–60 min segments of the filtered signals contain distinctively different frequency components. As shown in Figures 6A,B, wavelet analysis reveals an augmentation of the cardiac component (0.4–2 Hz) and an attenuation of the myogenic component (0.05–0.15 Hz) during 51–60 min as compared to the 1–10 min segment. This implies that when SBF data are sampled at 32 Hz, *S*_*E*_(*m, r, N*) is unable to reflect the structural properties of BFO with frequencies below 2 Hz. The difference in *S*_*E*_(*m, r, N*) between two segments of the original data is largely attributed to the components with frequencies higher than 2 Hz. In contrast, *S*_*E*_(*m, r*, τ, *N*) shows distinct differences between two segments for both the original data and filtered data, suggesting that this measure is able to reflect changes in structural properties of BFO below 2 Hz (Figure 6D).

**Figure 7:**
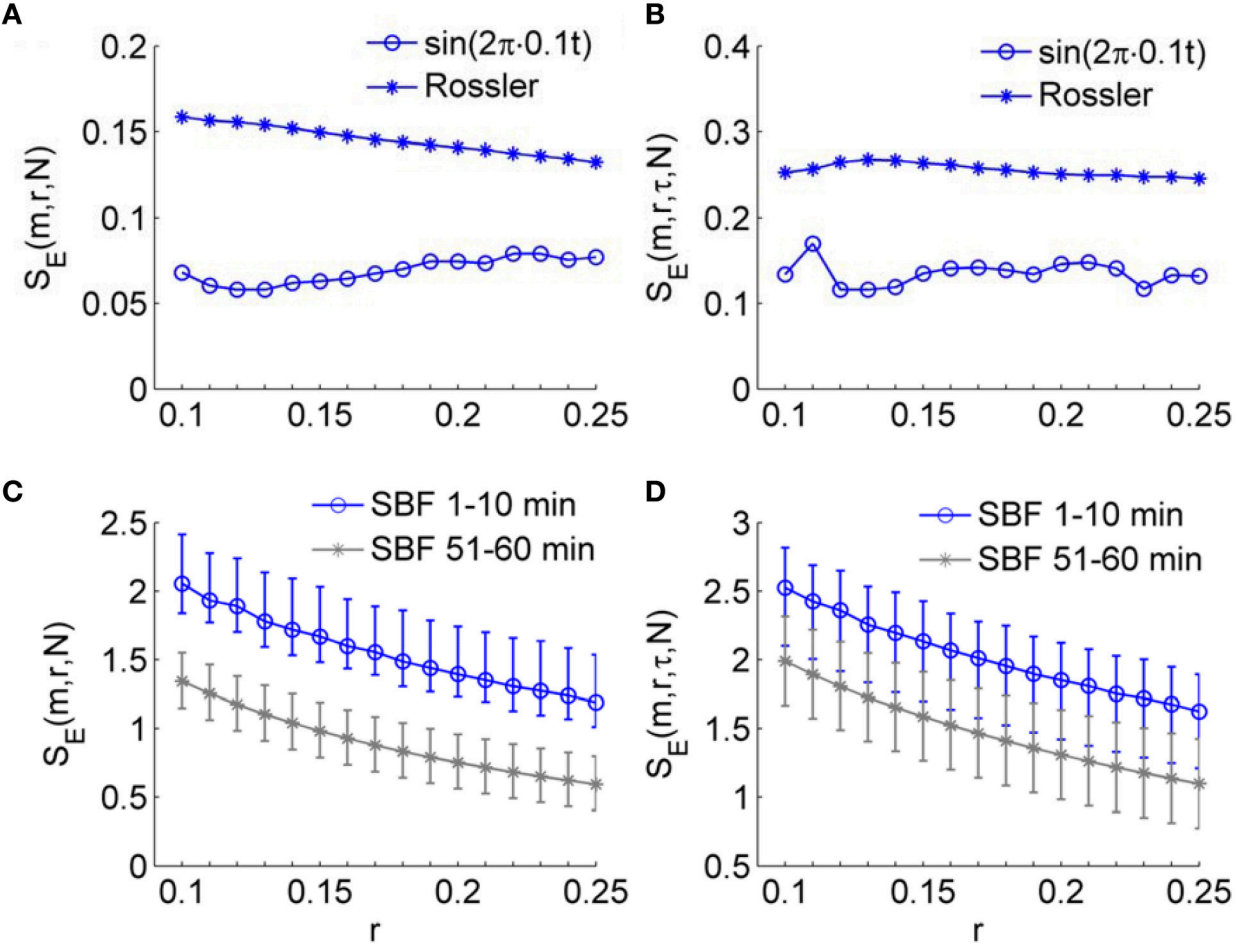
**Validation of the consistency of ***S***_***E***_(***m, r***, τ, ***N***) for varying parameter ***r*** and fixed parameter ***m*****. The sine wave, the variable *x*_1_ of Rössler attractor, and the SBF signals from all the subjects were used. All series were sampled at δ_*t*_=0.0313 s or *f*_*s*_ = 32 Hz and had a length of *N* = 4800. The parameters *m* = 3 and r between 0.1 and 0.25 were used. **(A)** *S*_*E*_(*m, r, N*) for the sine wave and Rössler attractor. **(B)** *S*_*E*_(*m, r*, τ, *N*) for the sine wave and Rössler attractor. **(C,D)** Values of *S*_*E*_(*m, r, N*) and *S*_*E*_(*m, r*, τ, *N*) for the SBF signals. The results are shown as means, 5 percentiles, and 95 percentiles. The larger range of *S*_*E*_(*m, r*, τ, *N*) values compared to that of *S*_*E*_(*m, r, N*) values is mainly due to the more distinct difference in *S*_*E*_(*m, r*, τ, *N*) values between the young and old groups.

An important expected feature of *S*_*E*_(*m, r*, τ, *N*) is relative consistency. It has been demonstrated that *S*_*E*_(*m, r, N*) shows relative consistency (Richman and Moorman, 2000). That is, for two time series *X*_1_and *X*_2_, if *S*_*E*_(*m*_1_, *r*_1_)(*X*_1_) ≤ *S*_*E*_(*m*_1_, *r*_1_)(*X*_2_), then *S*_*E*_(*m*_2_, *r*_2_)(*X*_1_) ≤ *S*_*E*_(*m*_2_, *r*_2_)(*X*_2_). This means that if the *S*_*E*_ value of *X*_1_ is lower than that of *X*_2_ for one pair of parameters *m* and *r*, it is expected to do so for all pairs of parameters. In most studies of sample entropy, values of *m* of 2 or 3 and values of *r* between 0.1 and 0.25 have been used (Richman and Moorman, 2000; Lake et al., 2002). The results presented in Figure 5 indicate that for sine wave, Rössler attractor, and SBF signals, *S*_*E*_(*m, r*, τ, *N*) holds relative consistency for varying parameter m and fixed parameter *r*. Figure 5 shows that *S*_*E*_(*m, r*, τ, *N*) value of the sine wave is lower than that of Rössler attractor for values of m between 2 and 7 and *r* = 0.2; Figure 6D show that *S*_*E*_(*m, r*, τ, *N*) of SBF data 1–10 min is higher than that of SBF data during 51–60 min for values of *m* = 2, 3 and *r* = 0.2. To examine whether *S*_*E*_(*m, r*, τ, *N*) shows consistency for varying values of *r* and fixed values of *m*, we calculated *S*_*E*_(*m, r*, τ, *N*) for the simulated signals and SBF data using the parameters *m* = 2, 3 and r from 0.1 to 0.25. We also calculated *S*_*E*_(*m, r, N*) of the same signals using the same parameters. Figure 7 shows the results for *m* = 3, which are similar to those for *m* = 2. Obviously, for both the simulated signals and SBF data, *S*_*E*_(*m, r*, τ, *N*) shows relative consistency for varying parameter *r* between 0.1 and 0.25 and fixed parameter *m*. It should be noted that the larger range of *S*_*E*_(*m, r*, τ, *N*) values compared to that of *S*_*E*_(*m, r, N*) values is mainly due to the more distinct difference in *S*_*E*_(*m, r*, τ, *N*) values between the young and old groups.

### Application of ***S***_***E***_(***m**, **r***, τ, ***N***) to SBF data

We applied the *S*_*E*_(*m, r*, τ, *N*) algorithm to the SBF data described earlier. Because P2/baseline shows significant difference between two groups (Figure 1B), we calculated *S*_*E*_(*m, r*, τ, *N*) for the SBF data during the baseline (1–10 min) and second peak (51–60 min). The parameters *m* = 3, *r* = 0.2, and τ = 12 were used. The reasons for choosing the parameters are as follows. First, for most of our data sets, when using 3 ≤ *m* ≤ 5, *S*_*E*_(*m, r*, τ, *N*) yields almost identical values (Figure 5C); when *m* > 5 the probabilities *C*^*m*^(*r*) and *C*^*m*+1^(*r*) (Equation 2) are too small that *S*_*E*_(*m, r*, τ, *N*) may become unreliable. Second, given a value of *m*, *S*_*E*_(*m, r*, τ, *N*) shows relative consistency for varying values of r (Figure 7D). That is, if *S*_*E*_(*m, r*, τ, *N*) of a data set is lower (higher) than that of another data set for a value of *r*, it will do so for other values of *r*. We therefore adopted a commonly used value *r* = 0.2. Third, the use of τ = 12 was based on the distribution of τ values for the SBF signals from all the subjects during the 1–10 min period (Figure 4D). Although the values of τ for the 51–60 min segments are smaller than those for the 1–10 min segments and are slightly different between two groups, we also used τ = 12 for the 51–60 min segments. We also calculated *S*_*E*_(*m, r, N*) for the SBF data using the same parameters *m*, *r*, and *N*.

To further understand the results of *S*_*E*_(*m, r*, τ, *N*), we performed wavelet analysis on the SBF signals during the 1–10 min and 51–60 min periods. The Morlet wavelet was used to implement continuous wavelet transforms. The relative wavelet amplitude of the metabolic (0.0095–0.02 Hz), neurogenic (0.02–0.05 Hz), myogenic (0.05–0.15 Hz), respiratory (0.15–0.4 Hz), and cardiac (0.4–2 Hz) components are defined as *A*_*f*1_, *f*_2_∕*A*_0.0095, 2_. Here, [*f*_1_, *f*_2_] is the frequency range of a specific frequency component, and *A*_*f*1_, *f*_2_ is the averaged absolute value of the wavelet transform over time and over the frequency range.

The Wilcoxon rank sum test was used to examine the difference between the young and old groups. All statistical tests were performed using SPSS 16 (SPSS, Chicago, IL) and the significant level was set at 0.05.

## Results

**Figure 8:**
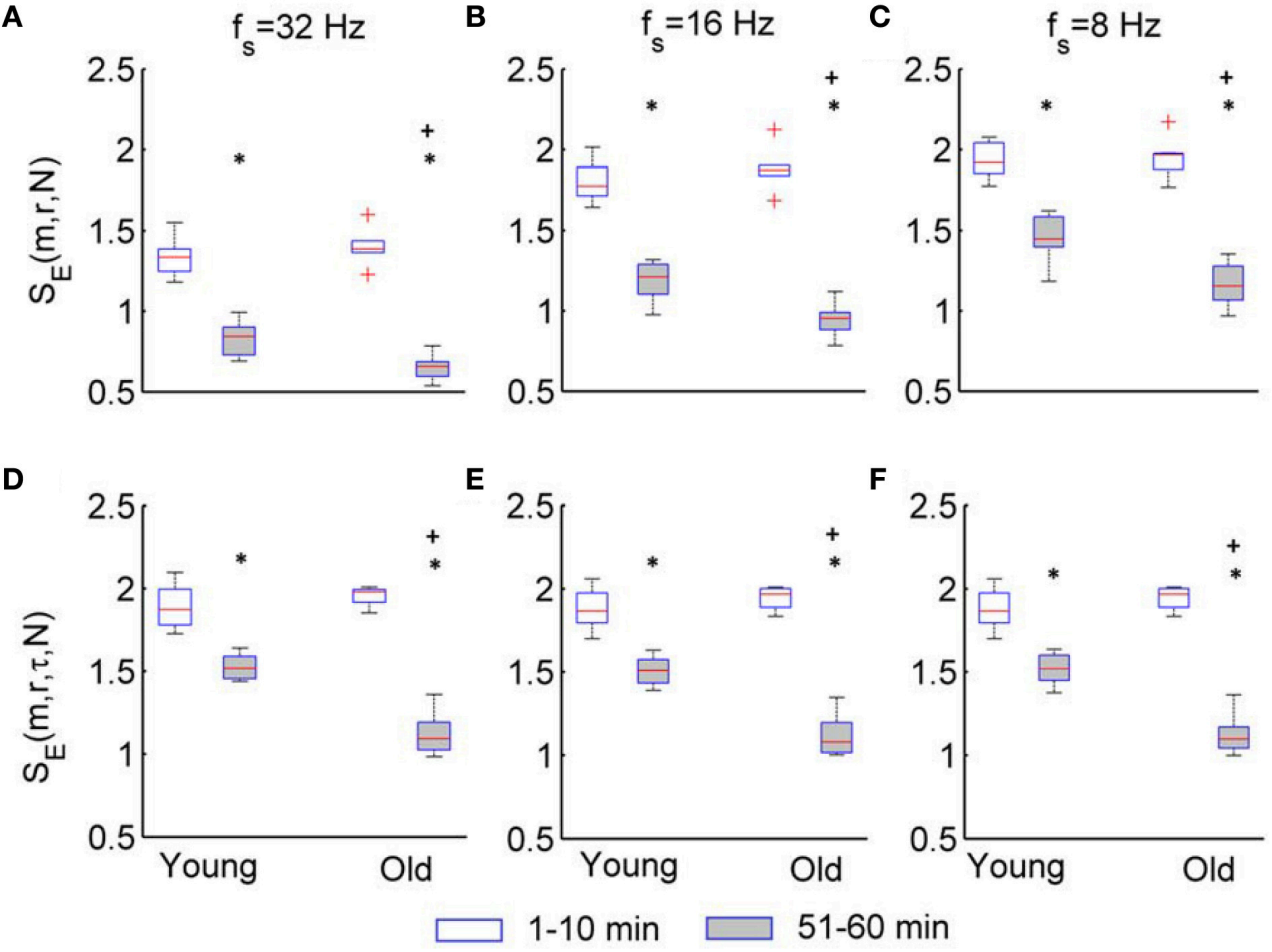
**Results of ***S***_***E***_(***m, r, N***) and ***S***_***E***_(***m, r***, τ, ***N***) for the two groups**. The parameters *m* = 3, *r* = 0.2, and τ = 12 were used. The stars indicate a significant difference in entropy between 1–10 min and 51–60 min period (*p* < 10^−3^, Wilcoxon signed rank test); the plus indicates a significant difference in entropy for 51–60 min period between two groups (*p* < 10^−3^, Wilcoxon rank sum test). **(A–C)** Results of *S*_*E*_(*m, r, N*) for *f*_*s*_ = 32, 16, and 8 Hz, respectively. **(D–F)** Results of *S*_*E*_(*m, r*, τ, *N*) for *f*_*s*_ = 32, 16, and 8 Hz, respectively.

Figure 8 compares the results of *S*_*E*_(*m, r*, τ, *N*) and *S*_*E*_(*m, r, N*) for two groups. For the sampling rate of 32, 16, and 8 Hz, *S*_*E*_(*m, r*, τ, *N*) shows almost identical results (Figures 8D–F). In both groups, *S*_*E*_(*m, r*, τ, *N*) of BFO during the second peak is significantly lower than that during the baseline (*p* < 10^−3^, Wilcoxon signed rank test). Although *S*_*E*_(*m, r*, τ, *N*) of BFO during the baseline does not show significant difference between two groups (*p* > 0.05), during the second peak it shows significantly lower values in older adults compared to young adults (*p* < 10^−3^).

Although *S*_*E*_(*m, r, N*) also shows significant differences between the baseline and second peak in two groups and between two groups during the second peak (Figures 8A–C), these differences depend on the sampling rate. Furthermore, these differences are likely attributed to changes of BFO fast than 2 Hz rather than changes in the characteristic frequencies associated with the underlying mechanisms of SBF regulation (see Figure 6).

**Figure 9:**
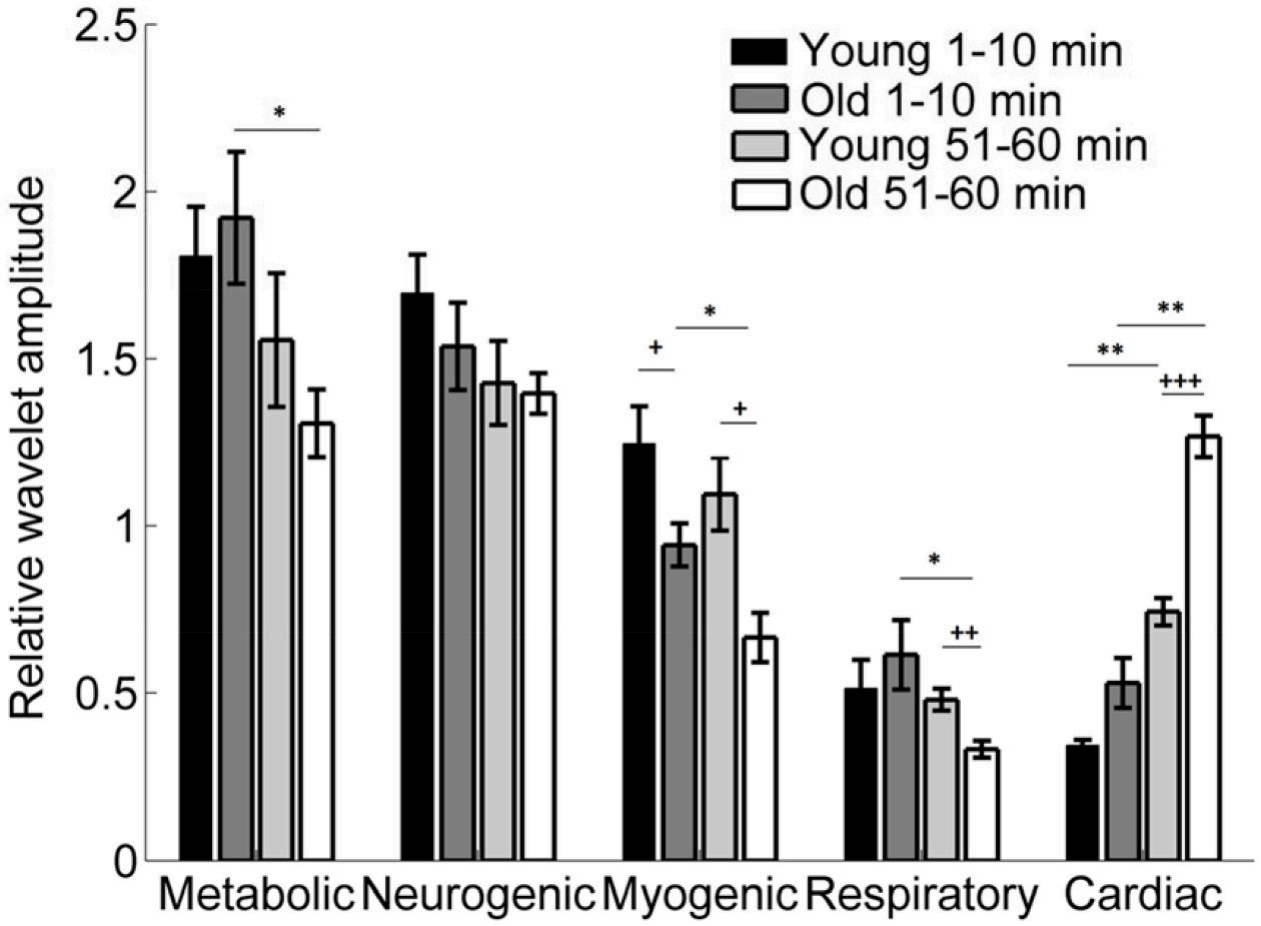
**Relative wavelet amplitudes of the metabolic (0.0095–0.02 Hz), neurogenic (0.02–0.05 Hz), myogenic (0.05–0.15 Hz), respiratory (0.15–0.4 Hz), and cardiac (0.4–2 Hz) components in two groups during the baseline (1–10 min) and second peak (51–60 min)**. ^*^p < 0.05, ^**^p < 10^−2^ (Wilcoxon signed rank test); ^+^*p* < 0.05, ^++^*p* < 10^−2^, ^+++^*p* < 10^−3^(Wilcoxon rank sum test).

Figure 9 shows the results of wavelet analysis of the SBF signals during the baseline and second peak. It is obvious that local heating induced a significant augmentation of the cardiac component in both group, particularly in old subjects, and a significant attenuation of the myogenic component.

## Discussion

In this paper, we ultilize a modified sample entropy approach for the assessment of SBF dynamics. The new measure reflects the degree of regularity of time series regardless of sampling rate, whereas the original sample entropy depends on the relationship between the frequency of the studied dynamics and sampling rate. We applied the new approach to SBF data from healthy young and older adults during local heating-induced second peak and observed a significant difference between the baseline and the second peak and between two groups.

The algorithm of *S*_*E*_ relies on the assumption that the vectors to be compared are independent of one another (Richman and Moorman, 2000). However, there might be many pairs of matched vectors that are dependent because of the correlation of the data and overlapping pairs of matches with points in common. As a direct consequence, *S*_*E*_ depends on sampling rate. In the modified *S*_*E*_ algorithm, any two successive data points of the vectors are separated by a lag τ. Our simulation results (Figure 3) suggest that the first minimum of MI(τ) is a more appropriate choice of the lag as compared to the first zero crossing of the autocorrelation function *C*(τ). Furthermore, we observed that for SBF signals the first zero crossing of *C*(τ) may yield a very large value or does not exist at all (Figure 4A). Thus, we chose the first minimum of MI(τ) as the lag. Because the lag in time is a constant, the first minimum of the MI function is usually proportional to the sampling rate. This feature ensures a constant degree of dependence between successive data points of the vectors to be compared when different sampling rates are used.

A great difficulty of analyzing BFO is the extremely low frequencies of the oscillatory components associated with the local control mechanisms of SBF. The characteristic frequency of the cardiac component (~1 Hz) is about 100 times the characteristic frequency of the endothelial-related metabolic activity (~0.01 Hz). Thus, any sampling rate that is appropriate for the cardiac component may be too high for the metabolic component. As we demonstrated earlier, when using a sampling rate of 32 Hz, *S*_*E*_(*m, r, N*) does not reflect changes of BFO with frequencies below 2 Hz (Figure 6C), whereas when using a lower sampling rate, e.g., 16 Hz or 8 Hz, changes of BFO below 2 Hz indeed result in changes in *S*_*E*_(*m, r, N*) because *S*_*E*_(*m, r, N*) and *S*_*E*_(*m, r*, τ, *N*) yield similar results when using a sampling rate of 8 Hz (Figures 8C,F). Therefore, when applying *S*_*E*_(*m, r, N*) to SBF data considerations need to be given as to what sampling rate should be used and cautions should be taken in interpreting the results. Our results show that the modified sample entropy, *S*_*E*_(*m, r*, τ, *N*), can yield consistent results regardless of sampling rate (Figures 5C, 8D–F).

**Figure 10:**
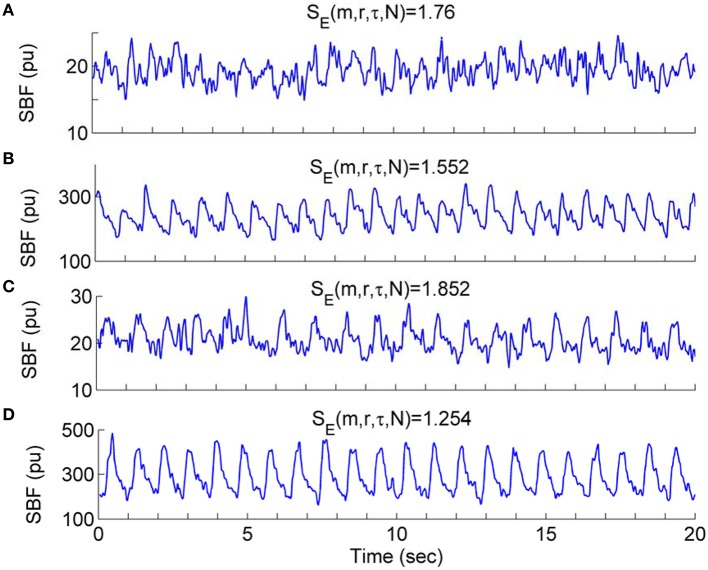
**Waveform profiles of SBF signal segments during the baseline and second peak**. The values of *S*_*E*_(*m, r*, τ, *N*) were calculated using the parameters *m* = 3, *r* = 0.2, τ = 12, and *N* = 4800. **(A,B)** SBF signal segments from a young subject during the baseline **(A)** and second peak **(B)**, respectively. **(C,D)** SBF signal segments from an old subject during the baseline **(C)** and second peak **(D)**, respectively.

Our results indicate that during the second peak (51–60 min) BFO were more regular compared to the baseline in both groups, especially for older adults, and that BFO were more regular in older adults compared to young adults (Figure 8). Using wavelet analysis, we observed that local heating induced a significant increase in energy of the cardiac component and a significant decrease in energy of the myogenic component in both groups (Figure 9) Furthermore, we observed more pronounced changes in energy of the cardiac and myogenic components in old adults. These results are consist with a study by Sheppard et al. (2011), in which local heating induced a significant absolute increase in spectral energy of all characteristic frequency components except the myogenic one and that the most pronounced increase in energy was that of the cardiac component. The authors suggested that this response was due to reduced resistance caused by vasodilation. These results can be intuitively understood by observing the SBF signal segments shown in Figure 10. Obviously, during baseline (Figures 10A,C) cardiac component was obscured by some components with higher frequencies; during the second peak, these high frequency components largely disappeared and thus the cardiac rhythm became more distinct, contributing to a decrease in entropy. On the other hand, during the second peak the cardiac frequency in the old subject seemed to become more regular as compared to that in the young subject. This further contributed to a lower value of entropy.

**Figure 11:**
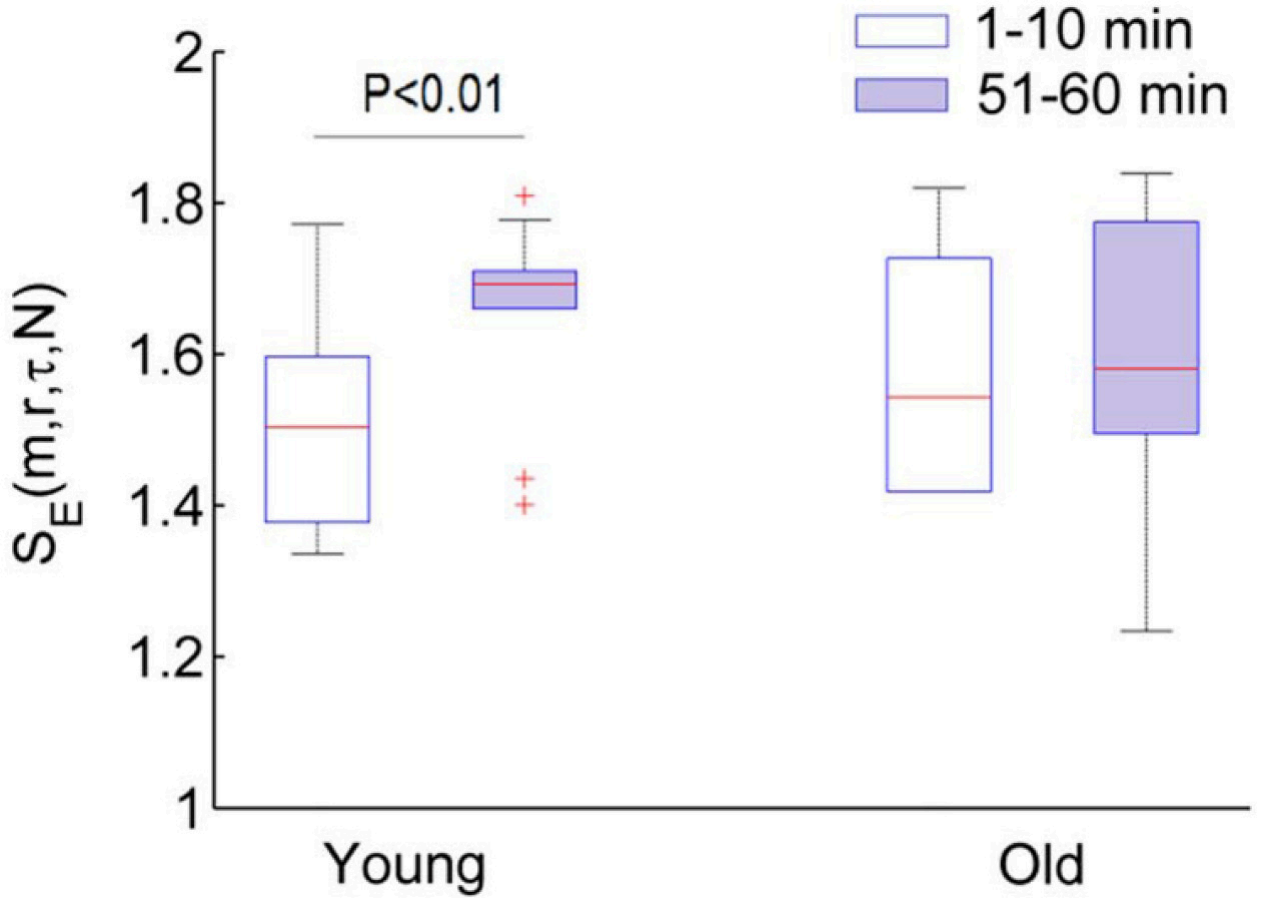
*****S***_***E***_(***m, r***, τ, ***N***) of SBF signals after removing the cardiac component**. The SBF signal (*f*_*s*_ = 32 Hz) was decomposed by utilizing a technique known as ensemble empirical mode decomposition and a new signal was reconstructed using the intrinsic mode functions excluding those with frequencies higher than 0.4 Hz. In the calculations of entropy, the parameters *m* = 3, *r* = 0.2, and τ = 12 were used. In young group, SBF signal after removing the cardiac component is more regular during the baseline (1–10 min) (Wilcoxon signed rank test).

To verify whether the more regular behavior of BFO during the second peak was attributed to enhanced cardiac oscillations, we performed the following experiment. The SBF signal was decomposed using a technique known as ensemble empirical mode decomposition {Wu and Huang, 2009 #103}. Then, a new signal was reconstructed from the intrinsic mode functions excluding those with frequencies higher than 0.4 Hz. Next, we calculated *S*_*E*_(*m, r*, τ, *N*) for the new signal using the same parameters, i.e., *m* = 3, *r* = 0.2, τ = 12. The results are shown in Figure 11. By comparing Figure 8D and Figure 11, it can be deduced that during the second peak the more regular behavior of BFO is mainly attributed to enhanced cardiac oscillations.

Vascular aging are characterized by functional and structural alterations of endothelium and smooth muscle cells that form the vessel wall, as well as alterations of the communication routes between these two cell layers {El Assar et al., 2013 #97}. It has been suggested that two main mechanisms are responsible for aging-related endothelia dysfunction: oxidative stress and inflammation {El Assar et al., 2013 #97}. Both increased oxidative stress and proinflammatory activity compromise NO bioavailability. In this study, we employed a fast local heating protocol to induce a biphasic blood flow response (Figure 1). It is generally accepted that the second peak is predominantly mediated by NO {Johnson and Kellogg, 2010 #96} {Minson, 2010 #11}. NO has direct effects on smooth muscle activity and possibly play a role in inhibiting vasoconstrictor response {Johnson and Kellogg, 2010 #96}. Our results indicate that local heating induced changes in both amplitude and structures of BFO. The power of the characteristic frequencies of BFO is commonly used to reflect the activities of the regulatory mechanisms {Stefanovska, 2007 #99}, whereas structural changes of BFO are often neglected. Our results indicate that in both groups more regular behaviors of BFO during the second peak were attributed to augmented cardiac component. After removing the cardiac component, SBF signal in young adults was more irregular during the second peak compared with the baseline (*p* < 0.01), while SBF signal in old adults showed similar degrees of regularity between the baseline and second peak (Figure 11). This is probably due to diminished interactions among the underlying mechanisms of SBF regulation in old adults.

Considering the results shown in Figures 8, 9, 11 and the waveform profiles presented in Figure 10, it seems that *S*_*E*_(*m, r*, τ, *N*) of SBF data is mainly attributed to high frequency components, i.e. the cardiac component. Because the characteristic frequency of the cardiac component (~1 Hz) is much higher than that of other components, e.g., metabolic and neurogenic components, any time scale that is appropriate for the cardiac component may be too short for observing oscillations of these components. Since SBF is regulated by multiple processes, each of which has its own temporal scale, to fully characterize the dynamics of BFO using the modified sample entropy, multiple time scales might need to be take into consideration.

## Conclusion

We modified the *S*_*E*_ algorithm by including a lag between successive data points of the vectors to be compared and the lag was chosen as the first minimum of the auto mutual information function of the time series. The new measure was able to reflect the degree of regularity of time series regardless of sampling rate where the original *S*_*E*_ cannot. We applied the new approach to SBF data from healthy young adults and older adults and observed a significant difference between the baseline and the maximal vasodilation periods and between young and older adults. BFO were more regular during the maximal vasodilation period compared to the baseline period in two groups and were more regular in older adults compared to young adults. However, the more regular behavior of BFO during the maximal vasodilation period was mainly attributed to augmented cardiac component. This study suggests that the modified SampEn approach may be useful for assessing microvascular function.

## Author contributions

Study concept and design: YJ. Acquisition of data: FL. Analysis and interpretation of data: FL, YJ. Drafting of manuscript: FL, YJ. Critical revision of manuscript for important intellectual content: FL, YJ. Obtained funding: YJ. Study supervision: YJ.

### Conflict of interest statement

The authors declare that the research was conducted in the absence of any commercial or financial relationships that could be construed as a potential conflict of interest.
